# Data on gene and protein expression changes induced by apabetalone (RVX-208) in ex vivo treated human whole blood and primary hepatocytes

**DOI:** 10.1016/j.dib.2016.07.047

**Published:** 2016-07-29

**Authors:** Sylwia Wasiak, Dean Gilham, Laura M. Tsujikawa, Christopher Halliday, Karen Norek, Reena G. Patel, Kevin G. McLure, Peter R. Young, Allan Gordon, Ewelina Kulikowski, Jan Johansson, Michael Sweeney, Norman C. Wong

**Affiliations:** a Resverlogix Corp., Calgary, Canada; b Resverlogix Corp., San Francisco, USA

**Keywords:** Bromodomain, BET proteins, BET inhibitor, RVX-208, JQ1, Vascular inflammation, ApoA-I, Apolipoprotein A-I, African green monkey, Primary human hepatocytes, Gene expression, Microarrays

## Abstract

Apabetalone (RVX-208) inhibits the interaction between epigenetic regulators known as bromodomain and extraterminal (BET) proteins and acetyl-lysine marks on histone tails. Data presented here supports the manuscript published in Atherosclerosis “*RVX-208, a BET-inhibitor for Treating Atherosclerotic Cardiovascular Disease, Raises ApoA-I/HDL and Represses Pathways that Contribute to Cardiovascular Disease”* (Gilham et al., 2016) [1]. It shows that RVX-208 and a comparator BET inhibitor (BETi) JQ1 increase mRNA expression and production of apolipoprotein A-I (ApoA-I), the main protein component of high density lipoproteins, in primary human and African green monkey hepatocytes. In addition, reported here are gene expression changes from a microarray-based analysis of human whole blood and of primary human hepatocytes treated with RVX-208.

**Specifications Table**

**Table:** 

Subject area	*Molecular biology*
More specific subject area	*Atherosclerosis*
Type of data	*Graphs and tables*
How data was acquired	*Real-time PCR using TaqMan assays; ELISA; Microarray analysis using Affymetrix Human Genome U133 Plus 2.0 and 2.4 Arrays.*
Data format	*Analyzed*
Experimental factors	*in vitro treatment of cultured primary cells with RVX-208, JQ1 or DMSO for up to 72 h.*
Experimental features	*mRNA and media were collected from cultured primary hepatocytes and analyzed by real-time PCR and ELISA, respectively. Human whole blood from healthy volunteers was treated ex vivo with BET inhibitors. Total RNA was extracted from treated whole blood and hepatocytes and analyzed using gene microarrays.*
Data source location	*51.010467°N, -114.123538°W*
Data accessibility	*Data is supplied with this article.*

**Value of the data**

• Data demonstrate suitability of human and African green monkey primary hepatocyte 3D culture systems for expression studies of the ApoA-I gene and protein.
• Data demonstrate suitability of a recently developed anti-proApoA-I antibody to measure newly produced ApoA-I protein in human primary hepatocytes.
• The gene expression data from human whole blood and primary hepatocytes reported here provide an RVX-208 transcriptional signature that can be compared to other compounds targeting BET proteins.

## 1 Data

Data presented here supports the manuscript published in Atherosclerosis “RVX-208, a BET-inhibitor for Treating Atherosclerotic Cardiovascular Disease, Raises ApoA-I/HDL and Represses Pathways that Contribute to Cardiovascular Disease” (Gilham et al., 2016) [1].

**Fig. 1:**
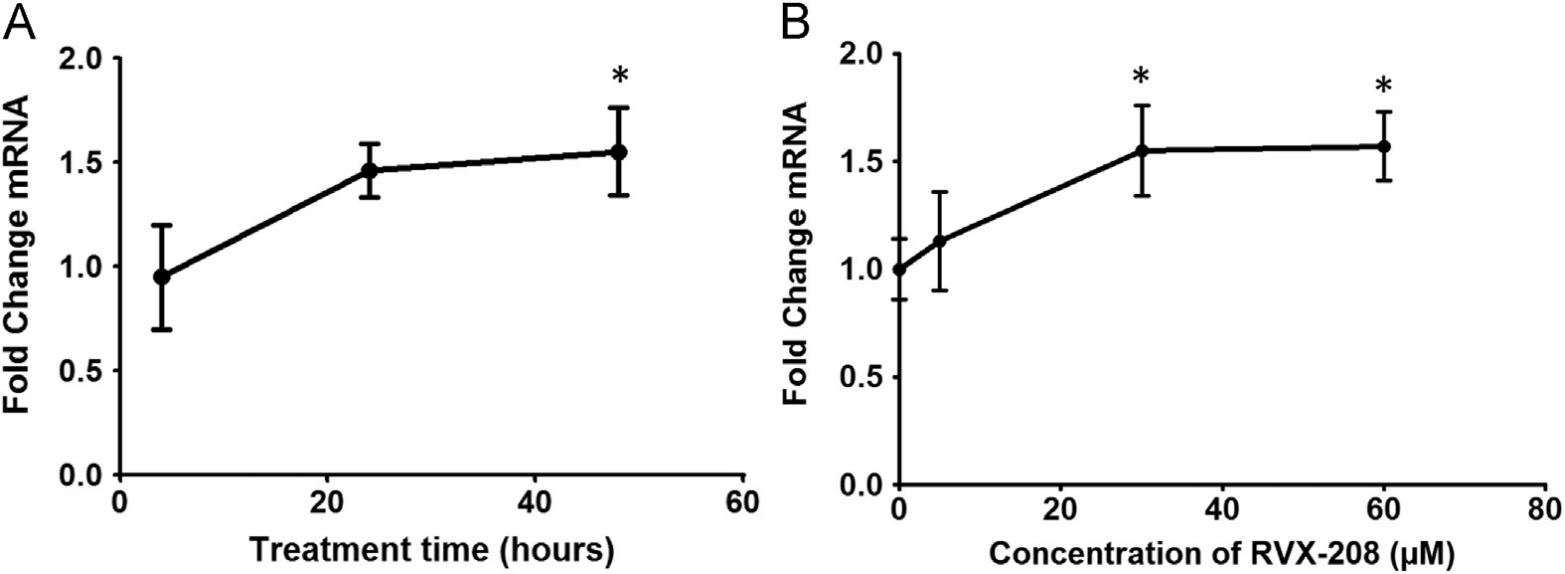
Effect of RVX-208 on ApoA-I mRNA expression in African green monkey hepatocytes. Hepatocyte 3-D cultures supplied by RegeneMed (San Diego, CA) were treated with 30 µM RVX-208 over a time course (A) or the indicated concentrations of RVX-208 for 48 h (B). Data are the mean from independent triplicate samples, while error bars represent standard deviation. ^⁎^*p*<0.05 versus DMSO treated samples at the same time point using two-tailed Student׳s *t*-tests.

**Fig. 2:**
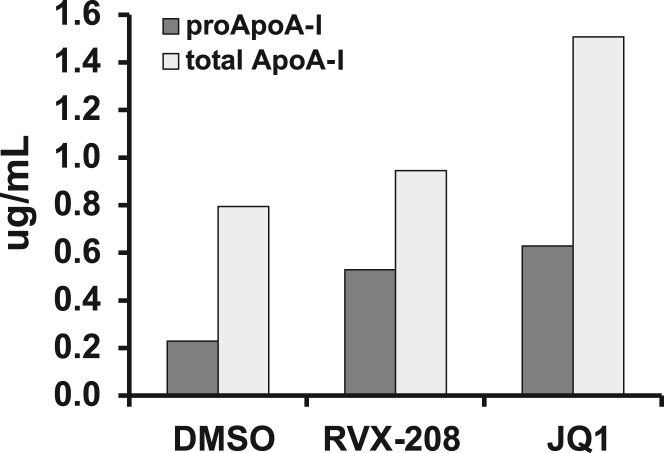
Comparison of effects of RVX-208 and JQ1 on ApoA-I protein secretion from cryopreserved primary human hepatocytes. Secreted total ApoA-I and proApoA-I protein levels were determined by ELISA in spent media from cells treated with 0.1% DMSO, 30 µM RVX-208 or 0.6 µM JQ1 for 72 h.

Combine with previous sentence. Expression of apolipoprotein A-I (ApoA-I) mRNA in response to RVX-208 treatment was assessed in primary hepatocytes from African green monkey grown in a 3-D culture system (Fig. 1). In primary human hepatocytes, the effect of RVX-208 on newly synthesized ApoA-I protein was compared to that of JQ1, a BETi with a distinct chemical scaffold (Fig. 2). Changes in expression of genes involved in inflammation and atherosclerosis [[2], [3], [4], [5], [6], [7], [8], [9], [10], [11], [12], [13], [14], [15], [16], [17], [18], [19], [20], [21], [22], [23], [24], [25], [26], [27], [28], [29], [30], [31], [32], [33], [34], [35], [36], [37], [38], [39], [40], [41], [42], [43], [44], [45], [46], [47], [48], [49], [50], [51], [52], [53], [54], [55], [56], [57], [58], [59], [60], [61], [62], [63], [64], [65], [66], [67], [68], [69], [70], [71], [72], [73], [74], [75], [76], [77], [78], [79]] were identified by microarrays from human whole blood and human primary hepatocytes treated in vitro with RVX-208 (Table 1, Table 2, Table 3, Table 4, Table 5).

## 2 Experimental design, materials and methods

### 2.1 Detection of ApoA-I mRNA in primary hepatocytes from African green monkey

Primary hepatocytes from African green monkey were supplied by RegeneMed Inc. (San Diego, CA). Stromal cells were grown concurrently on a nylon mesh scaffold with fresh liver parenchymal cells to create a three dimensional culture. Cells were treated with 0.1% DMSO or RVX-208 for 3 h, 24 h or 48 h, mRNA was purified with mRNA Catcher^TM^ PLUS kits (Life Technologies) and mRNA expression analysis was performed by TaqMan® based real-time PCR as described previously [80].

### 2.2 Detection of ApoA-I mRNA in primary human hepatocytes

Primary human hepatocytes (CellzDirect/Life Technologies) were plated as recommended by the supplier. Cells were treated with 0.1% DMSO, 30 µM RVX-208 or 0.6 µM JQ1 for 48 h, mRNA was purified and mRNA expression analyzed as above.

### 2.3 Detection of ApoA-I and proApoA-I secreted by primary human hepatocytes

Primary human hepatocytes (CellzDirect/Life Technologies) were treated with 0.1% DMSO, 30 µM RVX-208 or 0.6 µM JQ1 for 72 h and media samples containing secreted proteins were analyzed by ELISA. A rabbit monoclonal anti-proApoA-I antibody was generated using a synthetic peptide RHFWQQ_DEPP [1]. The antibody was used to coat EIA/RIA high binding surface microplates (Corning) overnight. Plates were washed, and then blocked with 5% skim milk. Recombinant poly-histidine tagged human proApoA-I (Genscript, Piscataway, NJ) was used as a standard. Recombinant protein or media samples with were introduced onto plates, incubated with anti-human ApoA-I (Calbiochem # 178470), and then with HRP conjugated anti-mouse IgG (Calbiochem # 401253). Color was developed by treatment with tetramethylbenzidine, followed by sulfuric acid. Plates were read on a Thermo Scientific Multiskan GO Spectrophotometer at 450 nm. ApoA-I ELISA was performed in a similar fashion as proApoA-I, except using the mouse anti-human ApoA-I antibody (Calbiochem # 178470). The standard was purified ApoA-I (Calbiochem # 178452) and it was detected using a polyclonal rabbit anti-human ApoA-I antibody (Calbiochem # 178422), followed by HRP conjugated anti-rabbit IgG (Calbiochem # 401353).

### 2.4 Gene expression microarray from human whole blood

After obtaining informed consent, whole blood was collected from three healthy volunteers into BD Vacutainer Sodium Heparin tubes (# 367874 ) and samples were inverted 10 times. Blood samples (1 mL) were combined with 1 mL of RPMI containing 2 mM glutamine, 1% penicillin/streptomycin, 20% FBS and 20 µM RVX-208 or vehicle (0.1% DMSO), followed by a 3 h or 24 h incubation at 37 °C in a tissue culture incubator (CO_2_ at 5.5% concentration). Treated samples were transferred to PAXgene RNA tubes (PreAnalytix/Qiagen), inverted 5 times and frozen. RNA was isolated with the PAXgene RNA kit according to the manufacturer׳s instructions. Microarrays were performed by Asuragen Inc. (Austin, TX) using the Affymetrix human U133 plus 2.4 Array. Gene expression changes were calculated as a fold change relative to DMSO treated samples. Genes with known roles in atherosclerosis, thrombosis or inflammation (based on published literature) and whose expression changed in response to RVX-208 treatment (*p*-value<0.05, Student׳s *t*-test) were compiled into pro-atherogenic and anti-atherogenic categories.

### 2.5 Gene expression microarray from primary human hepatocytes

Primary human hepatocytes (CellzDirect/Life Technologies) were plated in 24 well format at 500,000 cells/well, then overlaid with Matrigel™ as recommended by the supplier. Cells were treated with 0.1% DMSO or 30 µM RVX-208 for 48 h. Total RNA was extracted with the mirVana™ kit (Ambion) and sent to Asuragen Inc. (Austin, TX) for microarray analysis using Affymetrix Human Genome U133 Plus 2.0 Array. Gene expression changes were calculated as a fold change relative to DMSO treated cells. Genes encoding acute phase response proteins associated with HDL (based on http://homepages.uc.edu/~davidswm/HDLproteome.html) and whose expression changed in response to RVX-208 treatment (*p*-value<0.05, Student׳s *t*-test) were compiled.

## Figures and Tables

**Table 1:** The effect of RVX-208 on expression of pro-atherogenic genes in human whole blood treated ex vivo for 24 h.

**Gene symbol**	**Fold change**	**Effects on atherosclerotic processes in vitro and in vivo**	**Ref.**
CCL18	−7.0	pro-inflammatory cytokine; ↑ sites of occlusion during AMI	[4]
SPP1	−6.5	↓ atherosclerosis in SPP1/ApoE DKO with ANGII infusion; ↑ sites of occlusion during AMI	[4], [5]
CCL23	−5.8	mediates chemotaxis, expression of adhesion molecule and MMP-2 release from monocytes	[6]
PIK3R3	−5.1	PIK3R3 inhibitor ↓ atherosclerosis in ApoE KO; ↓ atherosclerosis in PIK3R3/LDLR DKO	[7], [8]
FCGR1A	−4.8	↓ atherosclerosis in FCGR1A/ApoE DKO	[9]
ITGA9	−4.6	enhances macrophage chemotaxis, receptor for SPP1	[10]
*IL2RA*	*−4.1*	*mAb against IL-2 ↓ atherosclerosis in ApoE KO; IL-2 stimulates T cells, but also expands Treg cells (atheroprotective)*	[11]
F13A1	−3.6	factor XIIIA inhibitor ↓ atherosclerosis in ApoE KO	[12]
PROK2	−3.4	pro-inflammatory; overexpressed at sites of aneurysm rupture	[13]
CXCL1	−3.2	↓ atherosclerosis in CXCL1/LDLR DKO	[14]
GHRL	−3.0	enhances monocyte adhesion and oxidized low-density lipoprotein binding	[15]
ANGPT1	−3.0	promotes monocyte and neutrophil migration and activates smooth muscle cells	[16]
LPL	−2.9	↓ atherosclerosis in LPL KO (BMT)/LDLR KO	[17]
IL26	−2.8	pro-inflammatory cytokine produced by Th17 cells	[18]
*C3*	*−2.5*	*↑ inflammation, destabilizes plaque; deficiency ↑atherogenesis in LDLR KO*	[19], [20]
IL23A	−2.0	cytokine, elevated in patients with peripheral arterial disease; role in advanced atherosclerotic plaque progression	[21], [22]
VEGFA	−2.0	↑ atherosclerosis in ApoE KO upon injection of recombinant VEGF	[23]
TLR2	−1.8	↓ atherosclerosis in TLR2/LDLR DKO	[24]
TNFSF13	−1.8	enhanced expression in atherosclerotic disorders	[25]
IL12RB1	−1.7	receptor for IL23A and IL12; mediates pro-inflammatory signaling	[26], [27]
TNFRSF8	−1.7	mediates activation and proliferation of T and B cells; altered TNFRSF8 function ↓ atherosclerosis in ApoE KO	[28]
NFAT5	−1.7	↓ atherosclerosis in NFAT5/apoE DKO and NFAT KO (BMT)/apoE KO	[29]
PIK3R2	−1.5	PI3K signaling promotes foam cell formation	[7], [8]
IL12B	−1.3	IL-12 induces T-cell recruitment into atherosclerotic plaque	[27]
OSMR	−1.3	monocyte- and T-cell-specific cytokine; promotes VSMC proliferation, migration and ECM protein synthesis	[30]
IL8	−1.3	pro-inflammatory cytokine; associated with AMI risk	[31]
AKR1B1	1.4	↑ atherosclerosis in AKR1B1 Tg/LDLR KO (diabetic)	[32]
PIK3R1	1.5	PI3K signaling promotes foam cell formation	[7], [8]
LTA	1.8	↓ atherosclerosis in LTA/ApoE DKO	[33]
IRAK4	1.8	↓ atherosclerosis in IRAK4 inactive knockin in ApoE KO plus carotid ligation	[34]
*C5*	*3.0*	*↑ inflammation, destabilizes plaque; ↑ atherosclerosis in C5/ApoE DKO*	[35], [36]
ADRB1	3.6	inhibitors attenuate atherosclerosis in ApoE KO	[37]

AMI: acute myocardial infarction; ANGII: angiotensin II; BMT: bone marrow transplant; DKO: double knockout; ECM: extracellular matrix; KO: knockout; mAb: monoclonal antibody; Tg: transgene; Treg: regulatory T-cells; VSMC: vascular smooth muscle cells; Italics indicates literature support for both pro-and anti-atherosclerotic roles. Fold change indicates changes in gene expression relative to 1 in vehicle-treated samples. For all entries, p<0.05 versus DMSO treated samples in a two-tailed Student׳s *t*-test.

**Table 2:** The effect of RVX-208 on expression of pro-atherogenic genes in human whole blood treated ex vivo for 3 h.

**Gene symbol**	**Fold change**	**Effects on atherosclerotic processes in vitro and in vivo**	**Ref.**
FN1	−21.0	FN1 promotes thrombogenesis and atherogenesis; ↑ sites of occlusion during AMI	[4], [38], [39], [40]
CCL2	−8.2	promotes chemotaxis in monocytes and basophils; binds to CCR2 and CCR4; ↑ sites of occlusion during AMI	[4], [41]
CCL8	−6.1	Promotes chemotaxis in monocytes, lymphocytes, basophils and eosinophils	[41]
CCL7	−4.8	promotes chemotaxis in monocytes and basophils; binds to chemokine receptors CCR1, CCR2, CCR3	[42]
SPP1	−3.1	↓ atherosclerosis SPP1/ApoE DKO with ANGII infusion; ↑ sites of occlusion during AMI	[4], [5]
ANGPT1	−3.1	promotes monocyte and neutrophil migration; activates VSMC	[16]
*THBS1*	*−2.9*	*Pro- and anti-atherogenic activities*	[43]
CXCL2	−2.7	chemokine involved in monocyte recruitment to the endothelium	[44], [45]
CXCL3	−2.5	chemokine involved in monocyte recruitment to the endothelium	[45]
CXCL1	−2.4	pro-inflammatory; promotes chemotaxis in neutrophils.	[45], [46]
CCR2	−2.4	chemokine receptor involved in monocyte recruitment; induces VSMC	[42], [47]
DDR1	−2.3	↓ atherosclerosis in DDR1/LDLR DKO	[48], [49]
ANGPTL3	−1.6	↓ atherosclerosis in ANGPTL3hypl/ApoE KO	[50]
ADORA2A	−1.5	↓ atherosclerosis in ADORA2A KO (BMT)/ApoE KO	[51]
IL8	−1.4	pro-inflammatory cytokine; associated with AMI risk	[31]
*CTSK*	*2.6*	*↓ atherosclerosis in CatK/ApoE DKO; stabilizes plaque*	[52]
TNFSF4	3.2	↓ atherosclerosis in ApoE/TNFSF4 DKO or ApoE KO+anti-TNFSF4 (MGP34) antibody-fed mice	[53]
ROCK2	3.3	↓ atherosclerosis in ROCK2 KO (BMT)/ApoE KO	[54]

AMI: acute myocardial infarction; ANGII: angiotensin II; BMT: bone marrow transplant; DKO: double knockout; ECM: extracellular matrix; hypl: recessive mutation; KO: knockout; mAb: monoclonal antibody; Tg: transgene; Treg: regulatory T-cells; VSMC: vascular smooth muscle cells. Italics indicates literature support for both pro-and anti-atherosclerotic roles. Fold change indicates changes in gene expression relative to 1 in vehicle-treated samples. For all entries, p<0.05 versus DMSO treated samples in a two-tailed Student׳s *t*-test.

**Table 3:** The effect of RVX-208 on expression of anti-atherogenic genes in human whole blood treated ex vivo for 24 h.

**Gene symbol**	**Fold change**	**Effects on atherosclerotic processes in vitro and in vivo**	**Ref.**
EDIL3	−3.3	regulates leukocyte-endothelial adhesion	[55]
IGF1	−2.3	↓ atherosclerosis in IGF1/ApoE DKO	[56], [57]
ADIPOQ	−2.0	↓ atherosclerosis in ADIPOQ OE/ApoE KO	[58]
ACVRL1	−1.8	Expressed in vascular endothelial cells and monocytes. Activation protects against atherosclerosis.	[59], [60]
COL18A1	−1.8	↓ atherosclerosis in endostatin (COL18A1 fragment)-fed ApoE KO	[61]
ACE2	−1.3	↑ atherosclerosis in ACE2-/y ApoE DKO and in ACE2-/y LDLR DKO	[62], [63]
TGFB1	1.4	↓ atherosclerosis and stabilizes plaques in TGFB1-overexpressing ApoE KO	[64]
TIMP1	1.6	↓ atherosclerosis and ↑ plaque stability in TIMP2 OE/ApoE KO; medial lamina ruptures in TIMP1-/y/ApoE DKO	[65], [66]
MERTK	1.6	↑ atherosclerosis in MERTK KO (BMT)/LDLR KO	[67], [68]
NR3C1	1.8	↓ vascular calcification without affecting atherosclerotic lesion in macrophage-specific NR3C1 KO (BMT)/LDLR KO	[69]
CDKN2A	1.9	↑ atherosclerosis in CDKN2A/ApoE DKO and in CDKN2A KO (BMT)/LDLR KO	[70], [71]
CXCR5	2.3	anti-inflammatory effects through monocyte signaling	[72]

BMT: bone marrow transplant; DKO: double knockout; KO: knockout; OE: overexpression; -/y : X-linked gene, 100% KO in males fold change indicates changes in gene expression relative to 1 in vehicle-treated samples. For all entries, p<0.05 versus DMSO treated samples in a two-tailed Student׳s *t*-test.

**Table 4:** Effect of RVX-208 on expression of anti-atherogenic genes in human whole blood treated ex vivo for 3 h.

**Gene symbol**	**Fold change**	**Effects on atherosclerotic processes in vitro and in vivo**	**Ref.**
IKBKB	−1.5	↑ atherosclerosis IKBKB KO (macrophage-specific)/ApoE KO	[73]
LIPA	−1.5	↓ atherosclerosis in LIPA-fed LDLRKO	[74]
IRF8	−1.3	↑ atherosclerosis in IRF8/ApoE DKO	[75]
*NR1H4*	*−1.3*	*↓ atherosclerosis in synthetic ligand-fed ApoE KO; ↓ atherosclerosis in NR1H4/ApoE DKO*	[76], [77]
ABCA1	1.5	↑ atherosclerosis in ABCA1/ApoE DKO	[78]
CXCL13	2.3	stabilizes plaque through CXCL13-CXCR5 interaction	[44], [79]

BMT: bone marrow transplant; DKO: double knockout; KO: knockout. Italics indicates literature support for both pro-and anti-atherosclerotic roles; fold change indicates changes in gene expression relative to 1 in vehicle-treated samples. For all entries, p<0.05 versus DMSO treated samples in a two-tailed Student׳s *t*-test.

**Table 5:** Genes that encode acute phase response proteins associated with HDL are modulated by RVX-208 in primary human hepatocytes. Gene expression changes measured in primary human hepatocytes treated with 30 µM RVX-208 for 48 h are expressed as fold change versus DMSO treated cells. For all values, p<0.05 as determined by a two-tailed Student׳s *t*-test.

**Gene name**	**Gene annotation**	**Fold change**	**Functional category**
complement component 9	C9	−9.3	Hemostasis
ceruloplasmin (ferroxidase)	CP	−5.4	Metal binding
lipopolysaccharide binding protein	LBP	−2.3	Immune response
alpha-2-HS-glycoprotein, fetuin A	AHSG	−2.1	Inflammation
complement component 1, s subcomponent	C1S	−2.0	Immune response
amyloid P component, serum	APCS	−2.0	Inflammation
inter-alpha-trypsin inhibitor heavy chain 2	ITIH2	−2.0	Proteolysis/inhibition/inflammation
coagulation factor II (thrombin)	F2	−1.8	Hemostasis
complement component 2	C2	−1.8	Immune response
alpha-2-macroglobulin	A2M	−1.8	Hemostasis
complement factor B	CFB	−1.6	Immune response
apolipoprotein H	APOH	−1.6	Hemostasis
haptoglobin	HP	−1.4	Inflammation
serum amyloid A2, A4	SAA1, SAA2, SAA4	−1.4	Lipid metabolism and transport

Microarray data from primary human hepatocytes treated with 30 µM RVX-208 for 48 h. Fold change versus DMSO treated cells is indicated.
