# Supplementary figures and images for: Data on gene and protein expression changes induced by apabetalone (RVX-208) in ex vivo treated human whole blood and primary hepatocytes
Source: Data Brief. 2016 Jul 29;8:1280–8. doi: 10.1016/j.dib.2016.07.047 (PMC4990638; doi:10.1016/j.dib.2016.07.047)

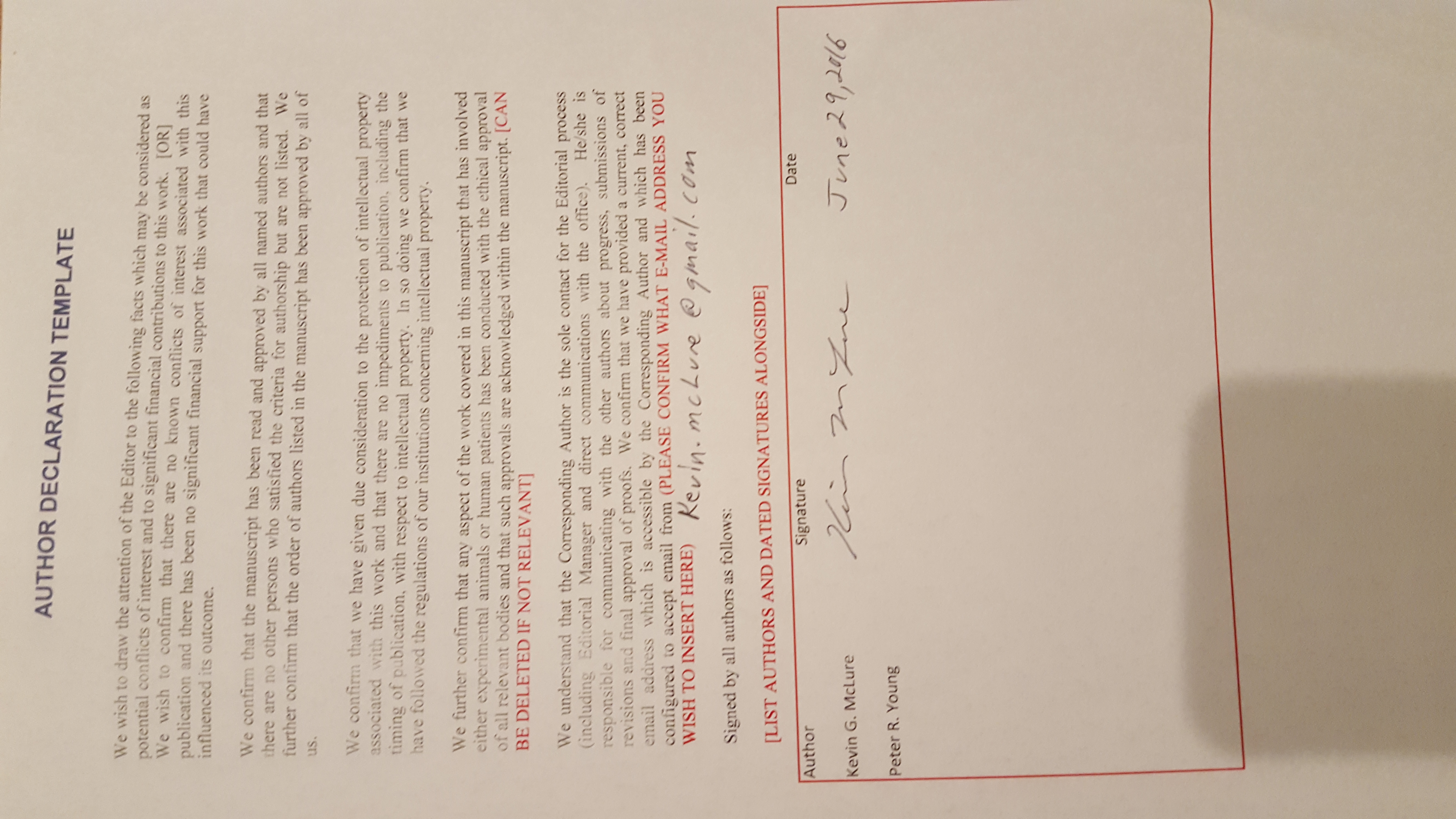

Supplement: Supplementary file 1 — Supplementary material. [file mmc1.zip › K.McLure_Author_Declaration_Form.jpg]
